# Supplementary material for: An Integrative Synthetic Biology Approach to Interrogating Cellular Ubiquitin and Ufm Signaling
Source: Int J Mol Sci. 2020 Jun 14;21(12):4231. doi: 10.3390/ijms21124231 (PMC7352202; doi:10.3390/ijms21124231)
Supplement: Supplementary file 1 [file ijms-21-04231-s001.pdf]

Supplementary Materials: An Integrative Synthetic Biology Approach to Interrogating Cellular Ubiquitin & Ufm Signaling

Chuanyin Li, Tianting Han, Rong Guo, Peng Chen, Chao Peng, Gali Prag and Ronggui Hu

Table S1. Plasmids used in this study.

Plasmid name	Construction method
pDEST32-UBE3A	LR reaction
pYESS-Ha-Ub-UBCH7-UBA1	Traditional cloning
pYESS-Ha-Ub-UBCH7-UBA1-UBE3A	Traditional cloning
pYESS-Ha-Ub-UBCH7-UBA1-UBE3A (C843A)	Traditional cloning
pYESS-SERPINB2-Flag-His6	B/P and L/R reactions
pYESS-ALDH1A2-Flag-His6	B/P and L/R reactions
pYESS-MCM6-Flag-His6	B/P and L/R reactions
pYESS-IL24-Flag-His6	B/P and L/R reactions
pYESS-CRP-Flag-His6	B/P and L/R reactions
pYESS-RAD23A-Flag-His6	B/P and L/R reactions
pYESS-PSMD4-Flag-His6	B/P and L/R reactions
pYESS-MSTO1-Flag-His6	B/P and L/R reactions
pDEST32-UFL1	LR reaction
pYESS-Ha-UFM1-UFC1-UBA5-UFL1	Traditional cloning
pYESS-DDRGK1-Flag-His6	B/P and L/R reactions
pYESS-MT1M-Flag-His6	B/P and L/R reactions
pYESS-TSC22D3-Flag-His6	B/P and L/R reactions
pET22b-UFC1-His6	Traditional cloning
pET22b-UBA5-His6	Traditional cloning
pGEX4T-1-UFL1	Traditional cloning
pET22b-UfSP2-His6	Traditional cloning
pET22b-His6-UFM1	Traditional cloning
pcDNA3.0-Myc-UBE3A	Traditional cloning
pcDNA3.0-Myc-UBE3A (C843A)	Site-directed mutagenesis
pcDNA3.0-SERPINB2-Flag	Traditional cloning
pcDNA3.0-ALDH1A2-Flag	Traditional cloning
pcDNA3.0-MCM6-Flag	Traditional cloning
pcDNA3.0-RAD23A-Flag	Traditional cloning

pcDNA3.0-PSMD4-Flag	Traditional cloning
pcDNA3.0-HA-UFM1	Traditional cloning
pcDNA3.0-UFL1-Myc	Traditional cloning
pcDNA3.0-UBA5-V5	Traditional cloning
pcDNA3.0-UFC1-V5	Traditional cloning
pcDNA3.0-DDRGK1-Flag	Traditional cloning
pcDNA3.0-TSC22D3-Flag	Traditional cloning
pcDNA3.0-MT1M-Flag	Traditional cloning
pcDNA3.0-MT1M (K31R)-Flag	Site-directed mutagenesis
PEGFP-UBE3A	Traditional cloning
PEGFP-UFL1	Traditional cloning
pcDNA3.1-SERPIB2-RFP	Traditional cloning
pcDNA3.1-DDRGK1-RFP	Traditional cloning
pcDNA3.1-MT1M-RFP	Traditional cloning
pcDNA3.1-TSC22D3-RFP	Traditional cloning
pRK5-His6-Ub	Traditional cloning
pRK5-Ha-Ub	Traditional cloning
pGEX4T-1-GST-UBE3A	Traditional cloning
pGEX4T-1-GST-UBE3A (C843A)	Site-directed mutagenesis
pET22b-UBCH7-His6	Traditional cloning
pET22b-His6-Ub	Traditional cloning
pET22b-UBA1-His6	Traditional cloning
pGL4.22-RARE-luciferase	Traditional cloning
pGL3-NF-κB-lucifearse	Traditional cloning
PRL-TK	Promega

Table S2. The antibodies used in this study.

Antibody	Source	Company	Catalog
UBE3A	M	Santa Cruz	sc-166689
Ubiquitin	M	Santa Cruz	sc-8017
ALDH1A2	R	Santa Cruz	sc-367527
SERPINB2	R	Proteintech	16035-1-AP
GAPDH	M	Proteintech	60004-1-Ig
Flag Tag	R	Proteintech	20543-1-AP
HA Tag	R	SIGMA	SAB4300603
His Tag	R	SIGMA	SAB1306085
Myc Tag	M	Santa Cruz	sc-40
UFL1	R	Proteintech	26087-1-AP
V5 tag	M	Proteintech	66007-1-Ig
Anti-Flag Affinity Gel	M	SIGMA	A4596
Anti-HA Affinity Gel	M	SIGMA	E6779

M， mouse ; R， Rabbit.

Table S3. qPCR primers used in this study.

Target Gene	Forward Primer (5′-3′)	Reverse Primer (5′-3′)
Gapdh	GGAGCGAGATCCCTCCAAAAT	GGCTGTTGTCATACTTCTCATGG
Hoxd4	CCCTCCGTGCGAGGAGTAT	GAAAGGCTGCTCACCGAAGT
Fgf8	GACCCCTTCGCAAAGCTCAT	CCGTTGCTCTTGGCGATCA

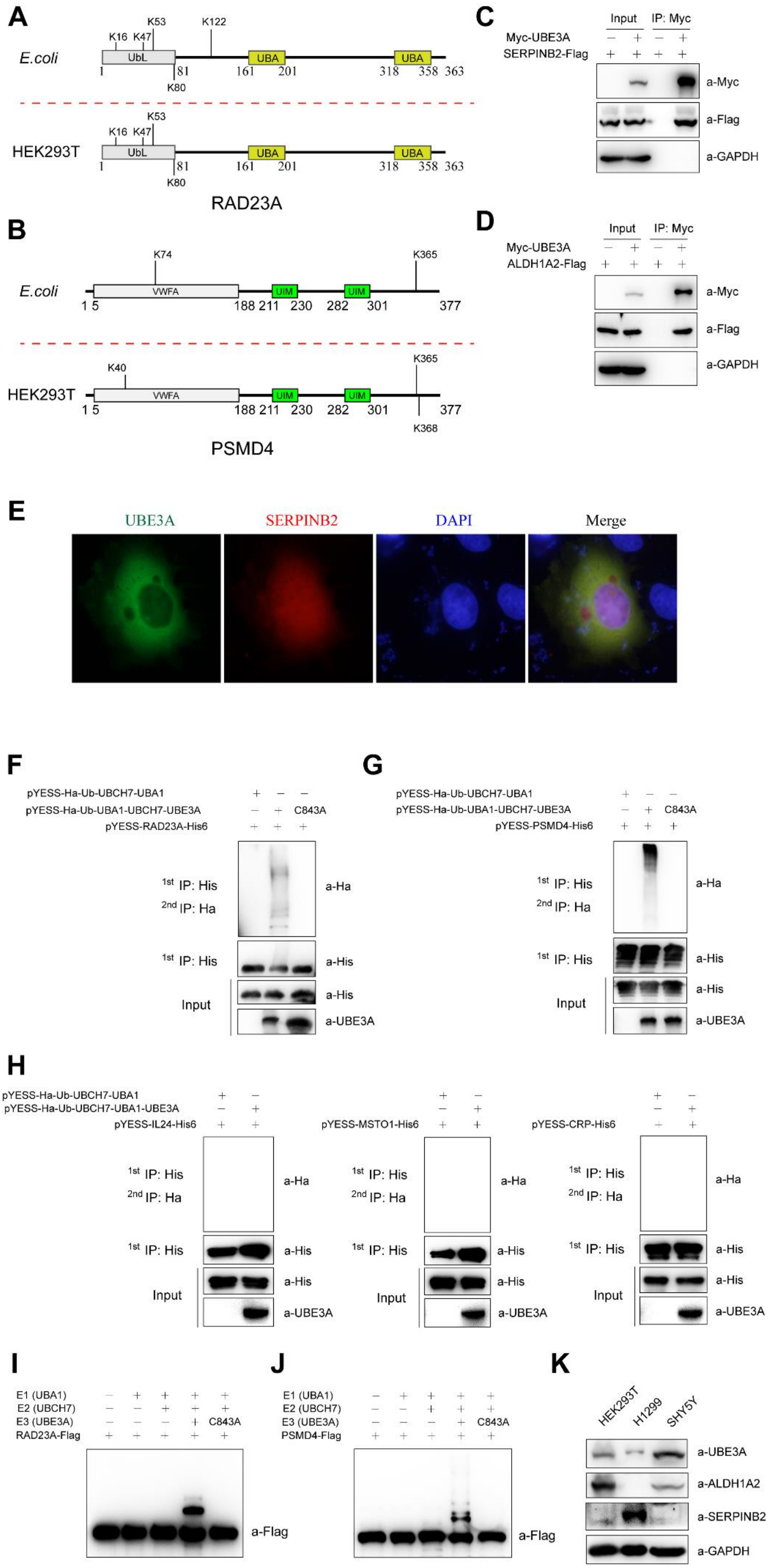

A
E.coli
HEK293T
UbL
UBA
K16 K47 K53 K80 K122
1 81 161 201 318 358 363
RAD23A
B
E.coli
HEK293T
VWFA
UIM
K74 K40 K365 K368
1 5 188 211 230 282 301 377
PSMD4
C
Input
IP: Myc
Myc-UBE3A
SERPINB2-Flag
a-Myc
a-Flag
a-GAPDH
D
Input
IP: Myc
Myc-UBE3A
ALDH1A2-Flag
a-Myc
a-Flag
a-GAPDH
E
UBE3A
SERPINB2
DAPI
Merge
F
pYESS-Ha-Ub-UBCH7-UBA1
pYESS-Ha-Ub-UBA1-UBCH7-UBE3A
pYESS-RAD23A-His6
C843A
1st IP: His
2nd IP: Ha
Input
a-Ha
a-His
a-UBE3A
G
pYESS-Ha-Ub-UBCH7-UBA1
pYESS-Ha-Ub-UBA1-UBCH7-UBE3A
pYESS-PSMD4-His6
C843A
1st IP: His
2nd IP: Ha
Input
a-Ha
a-His
a-UBE3A
H
pYESS-Ha-Ub-UBCH7-UBA1
pYESS-Ha-Ub-UBCH7-UBA1-UBE3A
pYESS-IL24-His6
pYESS-MSTO1-His6
pYESS-CRP-His6
1st IP: His
2nd IP: Ha
Input
a-Ha
a-His
a-UBE3A
I
E1 (UBA1)
E2 (UBCH7)
E3 (UBE3A)
RAD23A-Flag
C843A
a-Flag
J
E1 (UBA1)
E2 (UBCH7)
E3 (UBE3A)
PSMD4-Flag
C843A
a-Flag
K
HEK293T
H1299
SHY5Y
a-UBE3A
a-ALDH1A2
a-SERPINB2
a-GAPDH

Figure S1. The screening and validation of UBE3A substrates in 'YESS' system**.** (**A**) A comparison of the ubiquitination sites of RAD23A identified in *E. coli* ubiquitination system (upper panel) and mammalian HEK293T cells (lower panel). (**B**) The ubiquitination sites of PSMD4 identified in *E. coli* ubiquitination system (upper panel) and mammalian HEK293T cells (lower panel). (**C, D**) UBE3A-Myc and SERPINB2-Flag (**C**) orALDH1A2-Flag (**D**) could form complex in HEK293T cells. Cells were transfected with components indicated and immunoprecipitated with anti-Myc antibody, followed by immunoblotting analyses using indicated antibodies. (**E**) Co-localization of UBE3A with SERPINB2 as revealed by fluorescence microscopy analyses. HeLa cells were co-transfected with EGFP-UBE3A and SERPINB2-RFP， with nuclear DAPI staining. (**F**-**H**) Validation of UBE3A-mediated ubiquitination on RAD23A (**F**), PSMD4 (**G**), but not IL24, MSTO1 or CRP (**H**) in *E. coli* ubiquitination system. (**I, J**) In vitro ubiquitination assay of RAD23A(**I**) and PSMD4 (**J**) were carried out using the indicated recombinant proteins. (**K**) Detection the protein levels of UBE3A, ALDH1A2 or SERPINB2 in HEK293T, H1299 and SHY5Y cells.

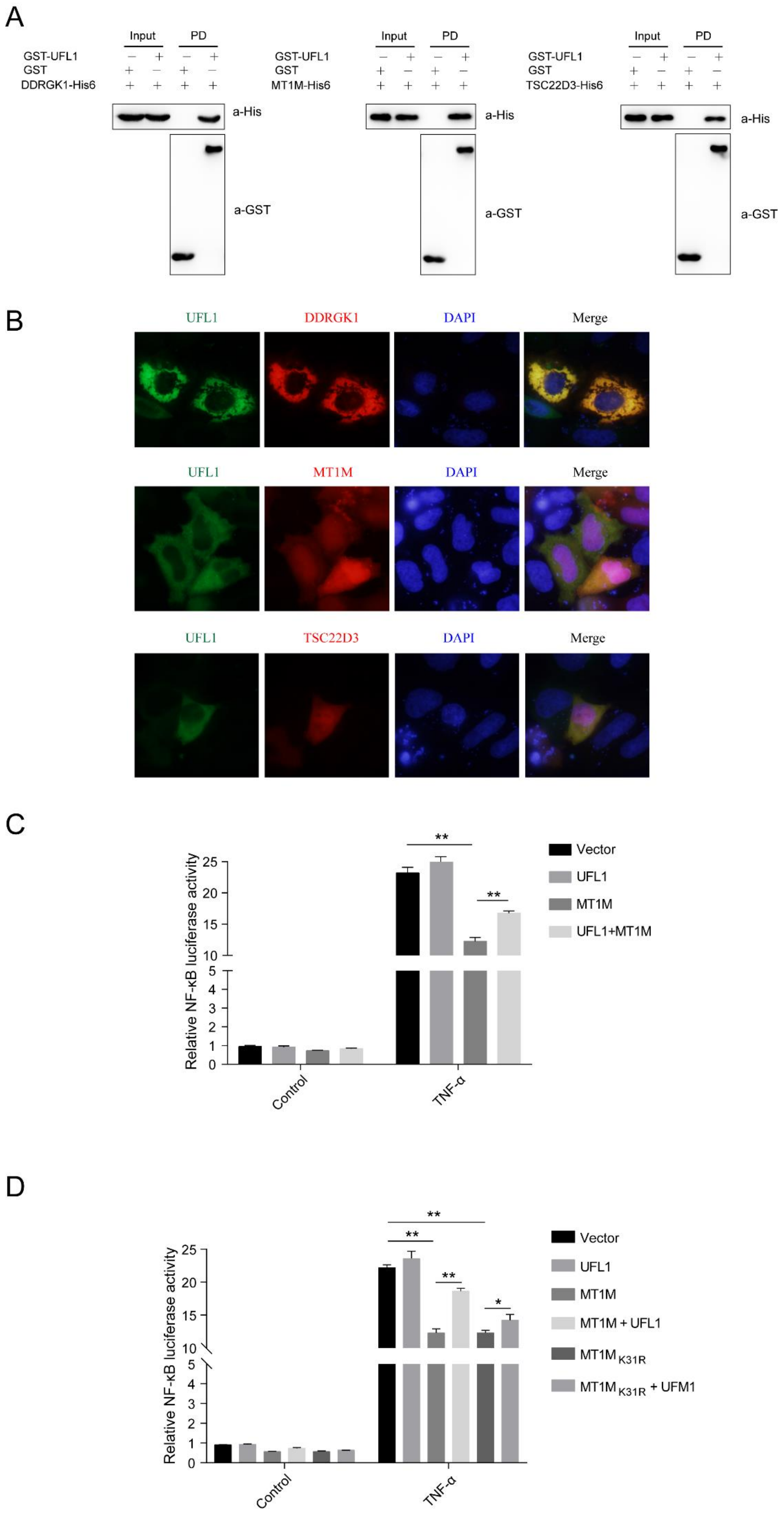
A
Input
PD
GST-UFL1
GST
DDRGK1-His6
a-His
a-GST
MT1M-His6
TSC22D3-His6
B
UFL1
DDRGK1
DAPI
Merge
MT1M
TSC22D3
C
Relative NF-κB luciferase activity
Control
TNF-α
Vector
UFL1
MT1M
UFL1+MT1M
D
MT1M + UFL1
MT1M K31R
MT1M K31R + UFM1

Figure S2. The screening and validation of UFL1 substrates in 'YESS' system. (**A**) Recombinant UFL1 directly interacted with DDRGK1, MT1M, or TSC22D3 in GST pulldown assays using the purified bacterially expressed proteins as indicated herein. PD, GST pulldown. (**B**) Co-localization of UFL1 with DDRGK1, MT1M or TSC22D3 as detected by fluorescence microscopy. HeLa cells were co-transfected with EGFP-UFL1 and DDRGK1/ MT1M/TSC22D3-RFP， with nuclear DAPI staining. (**C**) UFL1 disrupted the inhibitory effect of MT1M on TNF-α transactivated NF-κB luciferase activity. HEK293T cells were transfected with indicated plasmids and treated with or without TNF-α for 6 h before luciferase activity assays. *$p < 0.05$, significant difference; **$p < 0.01$, very significant difference. (**D**) UFL1-mediated ufmylation on K31 of MT1M partially disrupted the inhibition of MT1M on NF-κB luciferase activity. HEK293T cells were transfected with indicated plasmids and treated with or without TNF-α for 6 h before luciferase activity detected. *$p < 0.05$, significant difference; **$p < 0.01$, very significant difference.

A

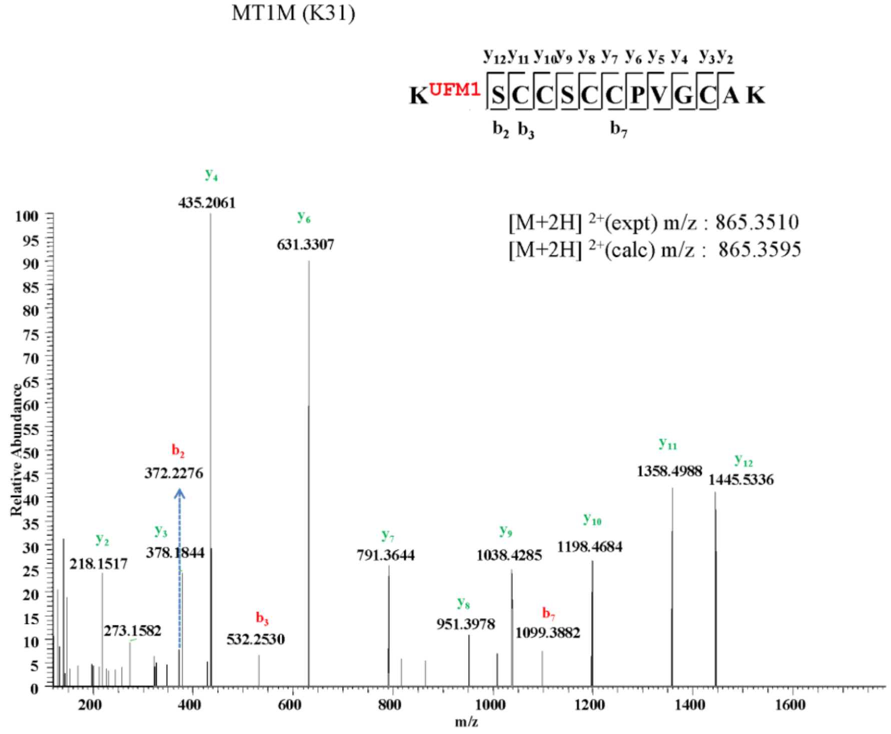

B

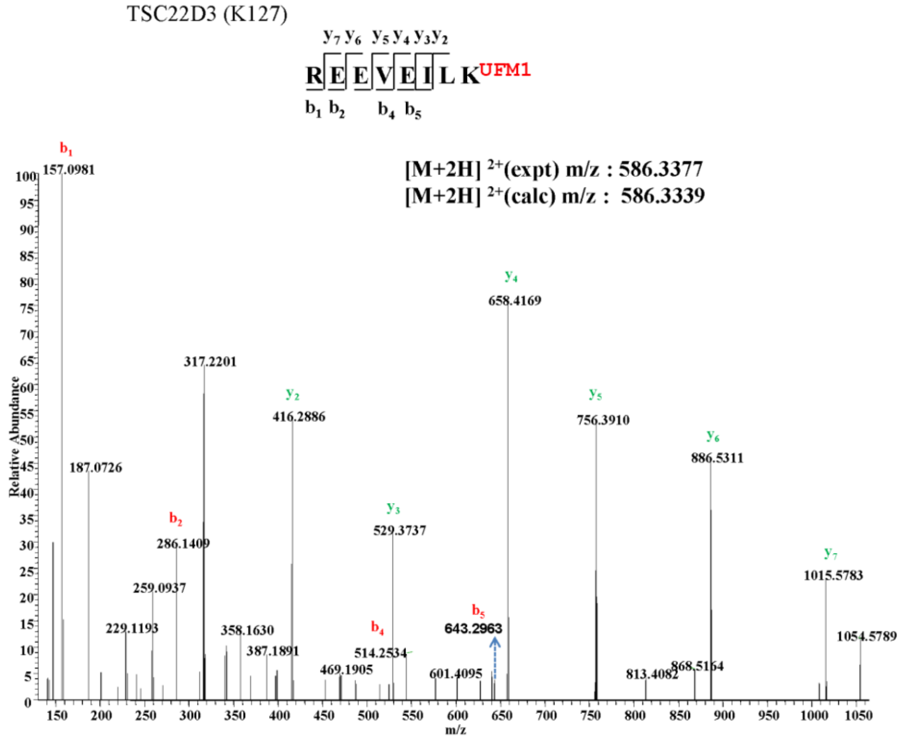

Figure S3. Mass spectrometry graphs for UFL1-mediated Ufmylation sites. Ufmylation sites included Lys[31] of MT1M(**A**), and Lys[127]of TSC22D3(**B**).
